# Westphalen’s diol diacetate: 19(10→5)-*abeo*-5β-cholest-9-ene-3β,6β-diyl diacetate

**DOI:** 10.1107/S1600536812045278

**Published:** 2012-11-10

**Authors:** Johana Ramírez Hernández, Jesús Sandoval-Ramírez, Socorro Meza-Reyes, José Luis Vega Báez, Sylvain Bernès

**Affiliations:** a Benemérita Universidad Autónoma de Puebla, Facultad de Ciencias Químicas, Ciudad Universitaria, Puebla, Pue. 72570, Mexico; b Universidad Autónoma de Nuevo León, UANL, Facultad de Ciencias Químicas, Av. Universidad S/N, Ciudad Universitaria, San Nicolás de los Garza, Nuevo León CP 66451, Mexico

## Abstract

The structure of the title steroid [alternative name: 3β,6β-diacet­oxy-5β-methyl-19-norcholest-9(10)-ene], C_31_H_50_O_4_, confirms the generally accepted mechanism for the rearrangement of a cholestan-5α-ol derivative reported a century ago by Westphalen. The methyl group at position 10 of the starting material migrates to position 5 in the steroidal nucleus, while a Δ^9^ bond is formed, as indicated by the C=C bond length of 1.347 (4) Å. The methyl transposition leaves the 5*R* configuration unchanged, with the methyl oriented towards the β face. During the rearrangement, the steroidal *B* ring experiences a conformational distortion from chair to envelope with the C atom at position 6 as the flap. In the title structure, the isopropyl group of the side chain is disordered over two positions, with occupancies of 0.733 (10) and 0.267 (10). The carbonyl O atom in the acetyl group at C3 is also disordered with an occupancy ratio of 0.62 (4):0.38 (4).

## Related literature

For the initial report on the Westphalen rearrangement, see: Westphalen (1915 ▶). For applications in steroid synthesis, see: Rodig *et al.* (1961 ▶); Knights & Hanson (2004 ▶); Pinto *et al.* (2008 ▶, 2009 ▶). For mechanistic aspects of this rearrangement, see: Kočovský & Černý (1977 ▶); Kočovský *et al.* (1979 ▶); Kamernitskii *et al.* (1987 ▶). 
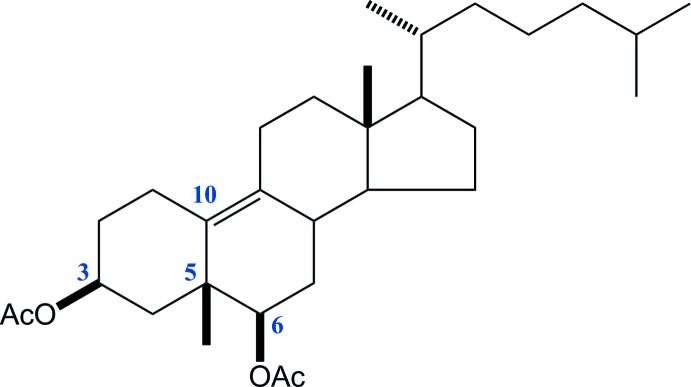


## Experimental

### 

#### Crystal data

C_31_H_50_O_4_
*M* *_r_* = 486.71
Orthorhombic, 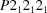

*a* = 9.2846 (15) Å
*b* = 10.7203 (18) Å
*c* = 29.982 (4) Å
*V* = 2984.2 (8) Å^3^
*Z* = 4
Mo *K*α radiation
μ = 0.07 mm^−1^
*T* = 298 K
0.6 × 0.5 × 0.5 mm

#### Data collection

Siemens P4 diffractometer
4350 measured reflections
3393 independent reflections
2827 reflections with *I* > 2σ(*I*)
*R*_int_ = 0.043
3 standard reflections every 97 reflections intensity decay: 0.5%

#### Refinement

*R*[*F* ^2^ > 2σ(*F* ^2^)] = 0.048
*wR*(*F* ^2^) = 0.136
*S* = 1.05
3393 reflections
364 parameters
57 restraints
H-atom parameters constrained
Δρ_max_ = 0.14 e Å^−3^
Δρ_min_ = −0.13 e Å^−3^

### 

Data collection: *XSCANS* (Siemens, 1996 ▶); cell refinement: *XSCANS*; data reduction: *XSCANS*; program(s) used to solve structure: *SHELXS97* (Sheldrick, 2008 ▶); program(s) used to refine structure: *SHELXL97* (Sheldrick, 2008 ▶); molecular graphics: *SHELXTL* (Sheldrick, 2008 ▶); software used to prepare material for publication: *SHELXL97*.

## Supplementary Material

Crystal structure: contains datablock(s) I, global. DOI: 10.1107/S1600536812045278/ff2087sup1.cif

Structure factors: contains datablock(s) I. DOI: 10.1107/S1600536812045278/ff2087Isup2.hkl

Additional supplementary materials:  crystallographic information; 3D view; checkCIF report

## Figures and Tables

**Fig. 1.**
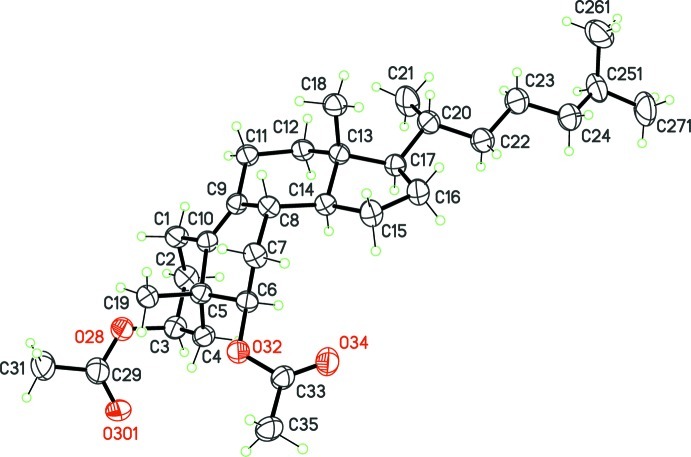
ORTEP view of the title molecule, with displacement ellipsoids for non-H atoms at the 30% probability level. For disordered parts, sites with occupancy less than 0.5 have been omitted.

## supplementary crystallographic information

### Comment

The Westphalen rearrangement is a useful synthetic tool in steroid chemistry, involving, for example, a 10β to 5β methyl shift, which has been used for the preparation of Δ^9^ steroids and 19-nor steroidal derivatives. It was originally applied in the cholestane series, and was further expanded, for instance, to 20-ketopregnanes (Rodig *et al.*, 1961), and more recently, to androstane (Knights & Hanson, 2004) and pregnane series (Pinto *et al.*, 2008, 2009). The mechanism of the rearrangement has been studied (Kočovský & Černý, 1977; Kočovský *et al.*, 1979), although it is not fully understood in a general case, because complex competing reaction pathways are in action (for a review on these mechanisms, see section 2 in Kamernitskii *et al.*, 1987, and references cited therein). A consensus has been reached, however, for products prepared under the Westphalen conditions in the cholestane series. Treatment with acetic anhydride and sulfuric acid promotes dehydration of the cholestan-5α-ol substrate, and the resulting carbocation is rearranged through the methyl 1,2-shift, before formation of the olefinic bond Δ^9^. The empty *p* orbital of the carbocation is electronically stabilized by the electron density of the O atom of the acetate at C6. With such a mechanism, a single epimer is expected as product, with the methyl at C5 oriented towards the β face. This stereochemistry has been confirmed in all the studied cases, but, surprisingly, the product of the very first report by Westphalen (1915) was never X-ray characterized.

During a study on the optimization of reaction conditions for the Westphalen rearrangement carried out on 5-hydroxy-5α-cholestane-3β,6β-diyl diacetate, which was the substrate used by Westphalen, we obtained in 80% yield the rearrangement product (see *Experimental*). The molecular structure (Fig. 1) and the absolute configuration for chiral centers are as expected. The saturated *A* and *C* rings have a chair conformation, while the *B* ring, which includes the C9═C10 double bond, is distorted to a half-chair conformation, close to an envelope with C6 as flap. Finally, the *D* ring adopts a half-chair conformation twisted on C13—C14. The observed conformation is indeed very similar to that described by Pinto *et al.* (2008) for the closely related derivative 3*β-*acetoxy-6β-hydroxy-5β-methyl-19-norcholest-9(10)-ene. An overlay between the steroidal nucleus of this structure and that of the title molecule gives a r.m.s. deviation limited to 0.156 Å, the largest deviation arising from the side chain. This group presents a degree of flexibility, as reflected by the disorder detected in the title compound for the isopropyl group. The same conformation for the *A*-*D* ring system was also observed for a Westphalen product in the pregnane series (Pinto *et al.*, 2009).

### Experimental

5-Hydroxy-5α-cholestane-3β,6β-diyl diacetate (6.1 g, 12.09 mmol) was treated with acetic anhydride (150 ml) for 20 min at 363 K. Then, NaHSO_4_ was added (1.74 g, 12.78 mmol) and stirred until complete consumption of the starting material (*ca*. 30 min). The mixture was poured in a 500 ml Erlenmeyer with pyridine and ice and vigorously stirred. The product, which deposited on the glass vessel, was dissolved in ethyl acetate, and then washed with saline solution, 5% HCl, distilled water, and finally 10% NaHCO_3_. This phase was dried over Na_2_SO_4_, and the solvent evaporated under reduced pressure. The crude (80% yield) was chromatographed over silicagel with petroleum ether and ethyl acetate (95:5) and crystallized from ethyl acetate. When KHSO_4_ was used, the Westphalen compound was obtained in 35% yield.

### Refinement

The isopropyl group in the lateral chain was found to be disordered over two positions, C251/C261/C271 and C252/C262/C272, and occupancies converged to 0.733 (10) and 0.267 (10), respectively. Both parts were restrained to have similar displacement parameters and geometry (*SIMU* and *SAME* restraints; Sheldrick, 2008). On the other side of the molecule, the carbonyl O atom in the acetyl group at C3 is also disordered over two sites, O301 and O302, with occupancies 0.62 (4) and 0.38 (4). All H atoms were placed in calculated positions and refined as riding to their carrier C atoms. C—H bond lengths were set to 0.96 (methyl) 0.97 (methylene) or 0.98 Å (methine), and isotropic parameters for H atoms were calculated as *U*_iso_(H) = *xU*_eq_(carrier C) with *x* = 1.5 (methyl) or *x* = 1.2 (methylene and methine groups). Friedel pairs (714) were merged, and absolute configuration assigned by fixing the configuration of known chiral centers.

### Crystal data

**Table:**

C_31_H_50_O_4_	*D*_x_ = 1.083 Mg m^−^^3^
*M**_r_* = 486.71	Melting point: 393 K
Orthorhombic, *P*2_1_2_1_2_1_	Mo *K*α radiation, λ = 0.71073 Å
Hall symbol: P 2ac 2ab	Cell parameters from 70 reflections
*a* = 9.2846 (15) Å	θ = 4.4–12.6°
*b* = 10.7203 (18) Å	µ = 0.07 mm^−^^1^
*c* = 29.982 (4) Å	*T* = 298 K
*V* = 2984.2 (8) Å^3^	Prism, colourless
*Z* = 4	0.6 × 0.5 × 0.5 mm
*F*(000) = 1072	

### Data collection

**Table:**

Siemens P4 diffractometer	*R*_int_ = 0.043
Radiation source: fine-focus sealed tube	θ_max_ = 26.2°, θ_min_ = 2.0°
Graphite monochromator	*h* = −11→1
ω scans	*k* = −13→1
4350 measured reflections	*l* = −37→1
3393 independent reflections	3 standard reflections every 97 reflections
2827 reflections with *I* > 2σ(*I*)	intensity decay: 0.5%

### Refinement

**Table:**

Refinement on *F*^2^	Secondary atom site location: difference Fourier map
Least-squares matrix: full	Hydrogen site location: inferred from neighbouring sites
*R*[*F*^2^ > 2σ(*F*^2^)] = 0.048	H-atom parameters constrained
*wR*(*F*^2^) = 0.136	*w* = 1/[σ^2^(*F*_o_^2^) + (0.0701*P*)^2^ + 0.3625*P*] where *P* = (*F*_o_^2^ + 2*F*_c_^2^)/3
*S* = 1.05	(Δ/σ)_max_ < 0.001
3393 reflections	Δρ_max_ = 0.14 e Å^−^^3^
364 parameters	Δρ_min_ = −0.13 e Å^−^^3^
57 restraints	Extinction correction: *SHELXL97* (Sheldrick, 2008), Fc^*^=kFc[1+0.001xFc^2^λ^3^/sin(2θ)]^-1/4^
0 constraints	Extinction coefficient: 0.0087 (15)
Primary atom site location: structure-invariant direct methods	

### Fractional atomic coordinates and isotropic or equivalent isotropic displacement parameters (Å^2^)

**Table:**

	*x*	*y*	*z*	*U*_iso_*/*U*_eq_	Occ. (<1)
C1	0.2670 (3)	0.4760 (3)	0.61696 (10)	0.0641 (7)	
H1A	0.2466	0.5401	0.5950	0.077*	
H1B	0.1814	0.4648	0.6351	0.077*	
C2	0.3009 (4)	0.3544 (3)	0.59299 (9)	0.0726 (8)	
H2A	0.2187	0.3299	0.5751	0.087*	
H2B	0.3821	0.3670	0.5731	0.087*	
C3	0.3362 (3)	0.2514 (3)	0.62582 (9)	0.0650 (7)	
H3A	0.3650	0.1761	0.6096	0.078*	
C4	0.4562 (3)	0.2908 (3)	0.65690 (9)	0.0606 (7)	
H4A	0.5454	0.2912	0.6401	0.073*	
H4B	0.4656	0.2278	0.6799	0.073*	
C5	0.4402 (3)	0.4198 (2)	0.68004 (8)	0.0527 (6)	
C6	0.5907 (3)	0.4565 (2)	0.69696 (8)	0.0532 (6)	
H6A	0.6594	0.4481	0.6723	0.064*	
C7	0.5959 (3)	0.5892 (2)	0.71400 (7)	0.0574 (6)	
H7A	0.6899	0.6063	0.7267	0.069*	
H7B	0.5240	0.6007	0.7371	0.069*	
C8	0.5666 (3)	0.6791 (2)	0.67539 (7)	0.0516 (6)	
H8A	0.5361	0.7588	0.6883	0.062*	
C9	0.4467 (3)	0.6345 (2)	0.64463 (7)	0.0507 (5)	
C10	0.3898 (3)	0.5190 (2)	0.64651 (8)	0.0514 (6)	
C11	0.4104 (3)	0.7329 (3)	0.61009 (8)	0.0585 (6)	
H11A	0.3727	0.8063	0.6250	0.070*	
H11B	0.3362	0.7012	0.5904	0.070*	
C12	0.5432 (3)	0.7699 (3)	0.58233 (8)	0.0586 (6)	
H12A	0.5173	0.8380	0.5626	0.070*	
H12B	0.5715	0.6996	0.5639	0.070*	
C13	0.6728 (3)	0.8100 (2)	0.61110 (8)	0.0517 (6)	
C14	0.6972 (3)	0.7048 (2)	0.64575 (7)	0.0513 (6)	
H14A	0.7129	0.6285	0.6285	0.062*	
C15	0.8427 (3)	0.7366 (3)	0.66716 (8)	0.0632 (7)	
H15A	0.8891	0.6625	0.6789	0.076*	
H15B	0.8314	0.7970	0.6910	0.076*	
C16	0.9295 (3)	0.7920 (3)	0.62790 (9)	0.0659 (7)	
H16A	1.0063	0.7356	0.6193	0.079*	
H16B	0.9718	0.8712	0.6365	0.079*	
C17	0.8231 (3)	0.8106 (3)	0.58856 (8)	0.0551 (6)	
H17A	0.8285	0.7350	0.5703	0.066*	
C18	0.6419 (3)	0.9375 (3)	0.63309 (10)	0.0661 (7)	
H18A	0.5566	0.9315	0.6511	0.099*	
H18B	0.6279	0.9994	0.6104	0.099*	
H18C	0.7220	0.9610	0.6515	0.099*	
C19	0.3317 (3)	0.4091 (3)	0.71907 (8)	0.0590 (6)	
H19A	0.3584	0.3405	0.7379	0.089*	
H19B	0.2367	0.3953	0.7074	0.089*	
H19C	0.3326	0.4849	0.7361	0.089*	
C20	0.8656 (3)	0.9204 (3)	0.55802 (9)	0.0666 (7)	
H20Z	0.8580	0.9974	0.5755	0.080*	
C21	0.7641 (4)	0.9322 (5)	0.51798 (12)	0.1005 (13)	
H21A	0.6682	0.9494	0.5283	0.151*	
H21B	0.7643	0.8555	0.5014	0.151*	
H21C	0.7960	0.9990	0.4991	0.151*	
C22	1.0228 (4)	0.9080 (3)	0.54214 (11)	0.0741 (8)	
H22A	1.0846	0.9012	0.5681	0.089*	
H22B	1.0321	0.8311	0.5253	0.089*	
C23	1.0756 (4)	1.0143 (4)	0.51364 (15)	0.0984 (12)	
H23A	1.0590	1.0918	0.5295	0.118*	
H23B	1.0186	1.0168	0.4866	0.118*	
C24	1.2325 (4)	1.0079 (4)	0.50092 (13)	0.0883 (10)	
H24A	1.2587	0.9211	0.4969	0.106*	0.733 (10)
H24B	1.2893	1.0402	0.5255	0.106*	0.733 (10)
H24C	1.2431	0.9432	0.4786	0.106*	0.267 (10)
H24D	1.2862	0.9814	0.5270	0.106*	0.267 (10)
C251	1.2727 (7)	1.0799 (5)	0.4585 (3)	0.088 (2)	0.733 (10)
H25A	1.2127	1.0454	0.4346	0.105*	0.733 (10)
C261	1.2338 (10)	1.2166 (6)	0.4617 (3)	0.116 (3)	0.733 (10)
H26A	1.1314	1.2249	0.4651	0.175*	0.733 (10)
H26B	1.2640	1.2587	0.4351	0.175*	0.733 (10)
H26C	1.2812	1.2528	0.4870	0.175*	0.733 (10)
C271	1.4250 (7)	1.0578 (8)	0.4448 (3)	0.127 (3)	0.733 (10)
H27A	1.4479	0.9711	0.4483	0.190*	0.733 (10)
H27B	1.4881	1.1070	0.4631	0.190*	0.733 (10)
H27C	1.4372	1.0812	0.4141	0.190*	0.733 (10)
C252	1.3037 (13)	1.1263 (15)	0.4829 (5)	0.076 (4)	0.267 (10)
H25B	1.2828	1.1972	0.5025	0.092*	0.267 (10)
C262	1.233 (4)	1.145 (4)	0.4374 (8)	0.192 (14)	0.267 (10)
H26D	1.1358	1.1140	0.4383	0.288*	0.267 (10)
H26E	1.2863	1.1010	0.4151	0.288*	0.267 (10)
H26F	1.2312	1.2325	0.4303	0.288*	0.267 (10)
C272	1.4604 (18)	1.109 (3)	0.4784 (16)	0.204 (14)	0.267 (10)
H27D	1.5024	1.0978	0.5073	0.306*	0.267 (10)
H27E	1.5016	1.1820	0.4646	0.306*	0.267 (10)
H27F	1.4795	1.0375	0.4602	0.306*	0.267 (10)
O28	0.2039 (2)	0.22733 (18)	0.65042 (8)	0.0734 (6)	
C29	0.1753 (4)	0.1143 (3)	0.66520 (13)	0.0832 (10)	
O301	0.2663 (9)	0.0327 (8)	0.6663 (4)	0.095 (3)	0.62 (4)
O302	0.228 (4)	0.0265 (18)	0.6439 (17)	0.186 (12)	0.38 (4)
C31	0.0398 (4)	0.1098 (4)	0.69085 (16)	0.1018 (12)	
H31A	0.0254	0.0269	0.7022	0.153*	
H31B	−0.0392	0.1320	0.6718	0.153*	
H31C	0.0451	0.1674	0.7153	0.153*	
O32	0.63096 (19)	0.36908 (19)	0.73216 (5)	0.0608 (5)	
C33	0.7731 (3)	0.3523 (3)	0.73874 (9)	0.0630 (7)	
O34	0.8643 (2)	0.4020 (3)	0.71683 (8)	0.0900 (8)	
C35	0.8011 (3)	0.2691 (3)	0.77742 (10)	0.0759 (8)	
H35A	0.9028	0.2546	0.7800	0.114*	
H35B	0.7524	0.1911	0.7730	0.114*	
H35C	0.7662	0.3079	0.8042	0.114*	

### Atomic displacement parameters (Å^2^)

**Table:**

	*U*^11^	*U*^22^	*U*^33^	*U*^12^	*U*^13^	*U*^23^
C1	0.0599 (16)	0.0645 (16)	0.0680 (16)	−0.0031 (14)	−0.0182 (13)	0.0092 (13)
C2	0.0761 (18)	0.0797 (19)	0.0621 (14)	−0.0114 (17)	−0.0165 (15)	−0.0051 (15)
C3	0.0641 (16)	0.0630 (16)	0.0680 (15)	−0.0010 (14)	−0.0006 (13)	−0.0091 (13)
C4	0.0592 (15)	0.0591 (15)	0.0635 (14)	0.0069 (13)	0.0008 (13)	0.0015 (12)
C5	0.0540 (13)	0.0563 (13)	0.0478 (11)	0.0030 (12)	−0.0023 (11)	0.0047 (11)
C6	0.0536 (13)	0.0625 (14)	0.0436 (11)	0.0021 (12)	−0.0039 (11)	0.0126 (11)
C7	0.0614 (14)	0.0685 (15)	0.0423 (10)	−0.0083 (13)	−0.0085 (11)	0.0050 (11)
C8	0.0563 (14)	0.0524 (13)	0.0462 (11)	−0.0029 (12)	−0.0032 (11)	0.0014 (10)
C9	0.0485 (12)	0.0577 (13)	0.0460 (11)	0.0033 (12)	−0.0038 (10)	0.0042 (11)
C10	0.0481 (12)	0.0595 (13)	0.0466 (11)	0.0009 (12)	−0.0067 (10)	0.0054 (11)
C11	0.0557 (14)	0.0617 (14)	0.0580 (13)	0.0024 (13)	−0.0094 (12)	0.0116 (12)
C12	0.0628 (15)	0.0628 (15)	0.0503 (12)	0.0024 (13)	−0.0088 (11)	0.0135 (12)
C13	0.0532 (13)	0.0533 (13)	0.0487 (11)	0.0003 (11)	0.0001 (11)	0.0031 (11)
C14	0.0545 (13)	0.0561 (13)	0.0431 (11)	−0.0013 (12)	−0.0045 (10)	0.0027 (10)
C15	0.0590 (15)	0.0795 (18)	0.0512 (12)	−0.0108 (15)	−0.0083 (11)	0.0059 (13)
C16	0.0588 (15)	0.0781 (18)	0.0608 (14)	−0.0087 (15)	−0.0020 (12)	0.0067 (14)
C17	0.0582 (14)	0.0583 (14)	0.0487 (11)	−0.0006 (12)	0.0014 (11)	0.0027 (11)
C18	0.0716 (17)	0.0560 (14)	0.0709 (15)	0.0020 (14)	0.0103 (15)	−0.0015 (13)
C19	0.0593 (15)	0.0602 (15)	0.0576 (13)	0.0002 (12)	0.0054 (12)	0.0065 (13)
C20	0.0692 (17)	0.0693 (16)	0.0614 (14)	−0.0007 (15)	0.0067 (14)	0.0113 (13)
C21	0.086 (2)	0.133 (3)	0.083 (2)	−0.017 (2)	−0.0076 (19)	0.054 (2)
C22	0.0759 (19)	0.0755 (19)	0.0708 (16)	0.0012 (16)	0.0130 (15)	0.0091 (16)
C23	0.085 (2)	0.093 (3)	0.116 (3)	0.004 (2)	0.036 (2)	0.030 (2)
C24	0.077 (2)	0.093 (2)	0.095 (2)	−0.0035 (19)	0.0192 (18)	0.014 (2)
C251	0.081 (3)	0.089 (4)	0.094 (4)	−0.031 (3)	0.022 (3)	−0.009 (3)
C261	0.131 (6)	0.089 (4)	0.129 (6)	−0.019 (4)	0.030 (5)	0.025 (4)
C271	0.085 (4)	0.140 (6)	0.156 (7)	−0.028 (4)	0.051 (5)	−0.004 (5)
C252	0.073 (7)	0.076 (8)	0.080 (8)	−0.024 (7)	−0.001 (7)	−0.014 (7)
C262	0.21 (3)	0.23 (3)	0.14 (2)	−0.06 (3)	0.01 (2)	0.07 (2)
C272	0.094 (13)	0.19 (3)	0.32 (4)	−0.067 (16)	0.03 (2)	0.03 (3)
O28	0.0671 (12)	0.0578 (11)	0.0954 (14)	−0.0046 (10)	0.0088 (11)	−0.0056 (11)
C29	0.082 (2)	0.0616 (18)	0.107 (2)	−0.0112 (18)	0.0187 (19)	−0.0178 (18)
O301	0.096 (4)	0.056 (4)	0.132 (6)	0.021 (3)	0.031 (4)	0.009 (3)
O302	0.22 (2)	0.100 (8)	0.24 (3)	−0.058 (10)	0.132 (19)	−0.068 (12)
C31	0.091 (2)	0.079 (2)	0.135 (3)	−0.015 (2)	0.029 (2)	−0.012 (2)
O32	0.0512 (9)	0.0758 (12)	0.0553 (9)	0.0015 (9)	−0.0064 (8)	0.0217 (9)
C33	0.0545 (15)	0.0722 (17)	0.0622 (14)	0.0075 (14)	−0.0023 (13)	0.0093 (14)
O34	0.0587 (11)	0.1137 (19)	0.0976 (15)	0.0029 (12)	0.0072 (11)	0.0390 (15)
C35	0.0668 (17)	0.087 (2)	0.0740 (16)	0.0147 (17)	−0.0089 (15)	0.0185 (16)

### Geometric parameters (Å, º)

**Table:**

C1—C10	1.516 (4)	C19—H19C	0.9600
C1—C2	1.521 (4)	C20—C21	1.532 (5)
C1—H1A	0.9700	C20—C22	1.541 (4)
C1—H1B	0.9700	C20—H20Z	0.9800
C2—C3	1.516 (4)	C21—H21A	0.9600
C2—H2A	0.9700	C21—H21B	0.9600
C2—H2B	0.9700	C21—H21C	0.9600
C3—O28	1.456 (3)	C22—C23	1.507 (4)
C3—C4	1.513 (4)	C22—H22A	0.9700
C3—H3A	0.9800	C22—H22B	0.9700
C4—C5	1.554 (4)	C23—C24	1.507 (5)
C4—H4A	0.9700	C23—H23A	0.9700
C4—H4B	0.9700	C23—H23B	0.9700
C5—C10	1.537 (3)	C24—C251	1.534 (7)
C5—C6	1.538 (4)	C24—C252	1.530 (14)
C5—C19	1.548 (3)	C24—H24A	0.9700
C6—O32	1.460 (3)	C24—H24B	0.9700
C6—C7	1.513 (4)	C24—H24C	0.9700
C6—H6A	0.9800	C24—H24D	0.9699
C7—C8	1.530 (3)	C251—C261	1.512 (8)
C7—H7A	0.9700	C251—C271	1.492 (8)
C7—H7B	0.9700	C251—H25A	0.9800
C8—C9	1.523 (3)	C261—H26A	0.9600
C8—C14	1.528 (3)	C261—H26B	0.9600
C8—H8A	0.9800	C261—H26C	0.9600
C9—C10	1.347 (4)	C271—H27A	0.9600
C9—C11	1.516 (3)	C271—H27B	0.9600
C11—C12	1.539 (4)	C271—H27C	0.9600
C11—H11A	0.9700	C252—C262	1.529 (17)
C11—H11B	0.9700	C252—C272	1.473 (17)
C12—C13	1.542 (4)	C252—H25B	0.9800
C12—H12A	0.9700	C262—H26D	0.9600
C12—H12B	0.9700	C262—H26E	0.9600
C13—C18	1.545 (4)	C262—H26F	0.9600
C13—C14	1.550 (3)	C272—H27D	0.9600
C13—C17	1.550 (3)	C272—H27E	0.9600
C14—C15	1.535 (3)	C272—H27F	0.9600
C14—H14A	0.9800	O28—C29	1.317 (4)
C15—C16	1.545 (4)	C29—O301	1.216 (7)
C15—H15A	0.9700	C29—O302	1.238 (17)
C15—H15B	0.9700	C29—C31	1.475 (5)
C16—C17	1.551 (4)	C31—H31A	0.9600
C16—H16A	0.9700	C31—H31B	0.9600
C16—H16B	0.9700	C31—H31C	0.9600
C17—C20	1.543 (4)	O32—C33	1.346 (3)
C17—H17A	0.9800	C33—O34	1.197 (3)
C18—H18A	0.9600	C33—C35	1.486 (4)
C18—H18B	0.9600	C35—H35A	0.9600
C18—H18C	0.9600	C35—H35B	0.9600
C19—H19A	0.9600	C35—H35C	0.9600
C19—H19B	0.9600		
			
C10—C1—C2	112.4 (2)	C16—C17—H17A	106.6
C10—C1—H1A	109.1	C13—C18—H18A	109.5
C2—C1—H1A	109.1	C13—C18—H18B	109.5
C10—C1—H1B	109.1	H18A—C18—H18B	109.5
C2—C1—H1B	109.1	C13—C18—H18C	109.5
H1A—C1—H1B	107.8	H18A—C18—H18C	109.5
C3—C2—C1	111.3 (2)	H18B—C18—H18C	109.5
C3—C2—H2A	109.4	C5—C19—H19A	109.5
C1—C2—H2A	109.4	C5—C19—H19B	109.5
C3—C2—H2B	109.4	H19A—C19—H19B	109.5
C1—C2—H2B	109.4	C5—C19—H19C	109.5
H2A—C2—H2B	108.0	H19A—C19—H19C	109.5
O28—C3—C4	111.0 (2)	H19B—C19—H19C	109.5
O28—C3—C2	106.0 (2)	C21—C20—C22	110.3 (3)
C4—C3—C2	110.8 (2)	C21—C20—C17	111.8 (3)
O28—C3—H3A	109.6	C22—C20—C17	111.1 (2)
C4—C3—H3A	109.6	C21—C20—H20Z	107.8
C2—C3—H3A	109.6	C22—C20—H20Z	107.8
C3—C4—C5	117.0 (2)	C17—C20—H20Z	107.8
C3—C4—H4A	108.1	C20—C21—H21A	109.5
C5—C4—H4A	108.1	C20—C21—H21B	109.5
C3—C4—H4B	108.1	H21A—C21—H21B	109.5
C5—C4—H4B	108.1	C20—C21—H21C	109.5
H4A—C4—H4B	107.3	H21A—C21—H21C	109.5
C10—C5—C6	108.4 (2)	H21B—C21—H21C	109.5
C10—C5—C19	110.4 (2)	C23—C22—C20	114.7 (3)
C6—C5—C19	111.16 (19)	C23—C22—H22A	108.6
C10—C5—C4	110.67 (19)	C20—C22—H22A	108.6
C6—C5—C4	106.7 (2)	C23—C22—H22B	108.6
C19—C5—C4	109.5 (2)	C20—C22—H22B	108.6
O32—C6—C7	110.59 (19)	H22A—C22—H22B	107.6
O32—C6—C5	107.9 (2)	C22—C23—C24	115.1 (3)
C7—C6—C5	112.4 (2)	C22—C23—H23A	108.5
O32—C6—H6A	108.6	C24—C23—H23A	108.5
C7—C6—H6A	108.6	C22—C23—H23B	108.5
C5—C6—H6A	108.6	C24—C23—H23B	108.5
C6—C7—C8	109.34 (18)	H23A—C23—H23B	107.5
C6—C7—H7A	109.8	C23—C24—C251	115.0 (4)
C8—C7—H7A	109.8	C23—C24—C252	118.0 (6)
C6—C7—H7B	109.8	C23—C24—H24A	108.5
C8—C7—H7B	109.8	C23—C24—H24B	108.5
H7A—C7—H7B	108.3	C23—C24—H24C	107.8
C9—C8—C14	106.51 (18)	C23—C24—H24D	107.8
C9—C8—C7	113.0 (2)	C251—C24—H24A	108.5
C14—C8—C7	114.4 (2)	C251—C24—H24B	108.5
C9—C8—H8A	107.6	C252—C24—H24C	107.8
C14—C8—H8A	107.6	C252—C24—H24D	107.8
C7—C8—H8A	107.6	H24A—C24—H24B	107.5
C10—C9—C11	125.5 (2)	H24C—C24—H24D	107.1
C10—C9—C8	123.4 (2)	C261—C251—C271	113.5 (6)
C11—C9—C8	111.0 (2)	C261—C251—C24	112.1 (6)
C9—C10—C1	123.4 (2)	C271—C251—C24	112.3 (6)
C9—C10—C5	123.0 (2)	C261—C251—H25A	106.1
C1—C10—C5	113.6 (2)	C271—C251—H25A	106.1
C9—C11—C12	111.7 (2)	C24—C251—H25A	106.1
C9—C11—H11A	109.3	C262—C252—C272	111.1 (19)
C12—C11—H11A	109.3	C262—C252—C24	103.8 (18)
C9—C11—H11B	109.3	C272—C252—C24	111.0 (15)
C12—C11—H11B	109.3	C262—C252—H25B	110.3
H11A—C11—H11B	107.9	C272—C252—H25B	110.3
C11—C12—C13	113.2 (2)	C24—C252—H25B	110.3
C11—C12—H12A	108.9	C252—C262—H26D	109.5
C13—C12—H12A	108.9	C252—C262—H26E	109.5
C11—C12—H12B	108.9	C252—C262—H26F	109.5
C13—C12—H12B	108.9	H26D—C262—H26E	109.5
H12A—C12—H12B	107.7	H26D—C262—H26F	109.5
C12—C13—C18	109.9 (2)	H26E—C262—H26F	109.5
C12—C13—C14	106.6 (2)	C252—C272—H27D	109.5
C18—C13—C14	112.64 (19)	C252—C272—H27E	109.5
C12—C13—C17	117.36 (19)	C252—C272—H27F	109.5
C18—C13—C17	110.5 (2)	H27D—C272—H27E	109.5
C14—C13—C17	99.4 (2)	H27D—C272—H27F	109.5
C8—C14—C15	119.70 (18)	H27E—C272—H27F	109.5
C8—C14—C13	113.9 (2)	C29—O28—C3	120.2 (3)
C15—C14—C13	104.3 (2)	O301—C29—O28	122.1 (5)
C8—C14—H14A	106.0	O302—C29—O28	116.5 (14)
C15—C14—H14A	106.0	O301—C29—C31	123.7 (5)
C13—C14—H14A	106.0	O302—C29—C31	125.5 (10)
C14—C15—C16	103.04 (19)	O28—C29—C31	112.2 (3)
C14—C15—H15A	111.2	C29—C31—H31A	109.5
C16—C15—H15A	111.2	C29—C31—H31B	109.5
C14—C15—H15B	111.2	H31A—C31—H31B	109.5
C16—C15—H15B	111.2	C29—C31—H31C	109.5
H15A—C15—H15B	109.1	H31A—C31—H31C	109.5
C15—C16—C17	107.3 (2)	H31B—C31—H31C	109.5
C15—C16—H16A	110.3	C33—O32—C6	116.3 (2)
C17—C16—H16A	110.3	O34—C33—O32	123.6 (3)
C15—C16—H16B	110.3	O34—C33—C35	124.9 (3)
C17—C16—H16B	110.3	O32—C33—C35	111.5 (2)
H16A—C16—H16B	108.5	C33—C35—H35A	109.5
C20—C17—C13	119.5 (2)	C33—C35—H35B	109.5
C20—C17—C16	112.7 (2)	H35A—C35—H35B	109.5
C13—C17—C16	103.94 (19)	C33—C35—H35C	109.5
C20—C17—H17A	106.6	H35A—C35—H35C	109.5
C13—C17—H17A	106.6	H35B—C35—H35C	109.5
			
C10—C1—C2—C3	57.8 (3)	C12—C13—C14—C8	−58.3 (3)
C1—C2—C3—O28	66.2 (3)	C18—C13—C14—C8	62.3 (3)
C1—C2—C3—C4	−54.4 (3)	C17—C13—C14—C8	179.29 (19)
O28—C3—C4—C5	−68.0 (3)	C12—C13—C14—C15	169.4 (2)
C2—C3—C4—C5	49.5 (3)	C18—C13—C14—C15	−70.0 (3)
C3—C4—C5—C10	−44.6 (3)	C17—C13—C14—C15	47.0 (2)
C3—C4—C5—C6	−162.4 (2)	C8—C14—C15—C16	−164.2 (2)
C3—C4—C5—C19	77.2 (3)	C13—C14—C15—C16	−35.3 (3)
C10—C5—C6—O32	174.13 (19)	C14—C15—C16—C17	9.6 (3)
C19—C5—C6—O32	52.7 (3)	C12—C13—C17—C20	79.2 (3)
C4—C5—C6—O32	−66.6 (2)	C18—C13—C17—C20	−47.9 (3)
C10—C5—C6—C7	51.9 (2)	C14—C13—C17—C20	−166.5 (2)
C19—C5—C6—C7	−69.5 (3)	C12—C13—C17—C16	−154.1 (2)
C4—C5—C6—C7	171.17 (19)	C18—C13—C17—C16	78.8 (3)
O32—C6—C7—C8	174.9 (2)	C14—C13—C17—C16	−39.8 (2)
C5—C6—C7—C8	−64.5 (3)	C15—C16—C17—C20	150.2 (3)
C6—C7—C8—C9	41.3 (3)	C15—C16—C17—C13	19.4 (3)
C6—C7—C8—C14	−80.8 (3)	C13—C17—C20—C21	−60.8 (3)
C14—C8—C9—C10	115.5 (3)	C16—C17—C20—C21	176.7 (3)
C7—C8—C9—C10	−10.9 (3)	C13—C17—C20—C22	175.5 (2)
C14—C8—C9—C11	−60.0 (3)	C16—C17—C20—C22	53.0 (3)
C7—C8—C9—C11	173.6 (2)	C21—C20—C22—C23	58.0 (4)
C11—C9—C10—C1	−7.3 (4)	C17—C20—C22—C23	−177.5 (3)
C8—C9—C10—C1	177.8 (2)	C20—C22—C23—C24	175.5 (3)
C11—C9—C10—C5	174.8 (2)	C22—C23—C24—C252	−164.3 (8)
C8—C9—C10—C5	0.0 (4)	C22—C23—C24—C251	156.2 (4)
C2—C1—C10—C9	128.1 (3)	C23—C24—C251—C261	57.6 (8)
C2—C1—C10—C5	−53.8 (3)	C252—C24—C251—C261	−46.2 (10)
C6—C5—C10—C9	−19.8 (3)	C23—C24—C251—C271	−173.3 (5)
C19—C5—C10—C9	102.2 (3)	C252—C24—C251—C271	82.9 (11)
C4—C5—C10—C9	−136.5 (3)	C23—C24—C252—C272	172 (2)
C6—C5—C10—C1	162.2 (2)	C251—C24—C252—C272	−94 (2)
C19—C5—C10—C1	−75.9 (3)	C23—C24—C252—C262	−69 (2)
C4—C5—C10—C1	45.5 (3)	C251—C24—C252—C262	25.6 (19)
C10—C9—C11—C12	−118.3 (3)	C4—C3—O28—C29	−91.3 (3)
C8—C9—C11—C12	57.1 (3)	C2—C3—O28—C29	148.3 (3)
C9—C11—C12—C13	−53.6 (3)	C3—O28—C29—O301	13.5 (9)
C11—C12—C13—C18	−70.6 (3)	C3—O28—C29—O302	−28 (3)
C11—C12—C13—C14	51.7 (3)	C3—O28—C29—C31	177.6 (3)
C11—C12—C13—C17	162.0 (2)	C7—C6—O32—C33	−82.4 (3)
C9—C8—C14—C15	−172.7 (2)	C5—C6—O32—C33	154.3 (2)
C7—C8—C14—C15	−47.2 (3)	C6—O32—C33—O34	−2.0 (4)
C9—C8—C14—C13	62.9 (3)	C6—O32—C33—C35	175.8 (2)
C7—C8—C14—C13	−171.6 (2)		
